# Reduction in Severity of All-Cause Gastroenteritis Requiring Hospitalisation in Children Vaccinated against Rotavirus in Malawi

**DOI:** 10.3390/v13122491

**Published:** 2021-12-13

**Authors:** Jonathan J. Mandolo, Marc Y. R. Henrion, Chimwemwe Mhango, End Chinyama, Richard Wachepa, Oscar Kanjerwa, Chikondi Malamba-Banda, Isaac T. Shawa, Daniel Hungerford, Arox W. Kamng’ona, Miren Iturriza-Gomara, Nigel A. Cunliffe, Khuzwayo C. Jere

**Affiliations:** 1 Virology Research Group, Malawi-Liverpool-Wellcome Trust Clinical Research Programme, Blantyre 312225, Malawi; jmandolo@medcol.mw (J.J.M.); mhenrion@mlw.mw (M.Y.R.H.); cmhango@mlw.mw (C.M.); endchinyama@gmail.com (E.C.); rwachepa@mlw.mw (R.W.); okanjerwa@mlw.mw (O.K.); cmalamba@mlw.mw (C.M.-B.); ishawa@medcol.mw (I.T.S.); awkamngona@kuhes.ac.mw (A.W.K.); 2 Department of Biomedical Sciences, School of Life Sciences and Health Professions, Kamuzu University of Health Sciences, Blantyre 312225, Malawi; 3 Department of Clinical Sciences, Liverpool School of Tropical Medicine, Liverpool L3 5QA, UK; 4 Centre for Global Vaccine Research, Institute of Infection, Veterinary and Ecological Sciences, University of Liverpool, Liverpool L69 7BE, UK; danhungi@liverpool.ac.uk (D.H.); miturrizagomara@path.org (M.I.-G.); nigelc@liverpool.ac.uk (N.A.C.); 5 Department of Medical Laboratory Sciences, School of Life Sciences and Health Professions, Kamuzu University of Health Sciences, Blantyre 312225, Malawi; 6 NIHR Health Protection Research Unit in Gastrointestinal Infections, University of Liverpool, Liverpool L69 7BE, UK; 7 Centre for Vaccine Innovation and Access, Program for Appropriate Technology in Health (PATH), 1218 Geneva, Switzerland

**Keywords:** rotavirus, genotypes, Malawi, gastroenteritis, severity scores

## Abstract

Rotavirus is the major cause of severe gastroenteritis in children aged <5 years. Introduction of the G1P[8] Rotarix^®^ rotavirus vaccine in Malawi in 2012 has reduced rotavirus-associated hospitalisations and diarrhoeal mortality. However, the impact of rotavirus vaccine on the severity of gastroenteritis presented in children requiring hospitalisation remains unknown. We conducted a hospital-based surveillance study to assess the impact of Rotarix^®^ vaccination on the severity of gastroenteritis presented by Malawian children. Stool samples were collected from children aged <5 years who required hospitalisation with acute gastroenteritis from December 2011 to October 2019. Gastroenteritis severity was determined using Ruuska and Vesikari scores. Rotavirus was detected using enzyme immunoassay. Rotavirus genotypes were determined using nested RT-PCR. Associations between Rotarix^®^ vaccination and gastroenteritis severity were investigated using adjusted linear regression. In total, 3159 children were enrolled. After adjusting for mid-upper arm circumference (MUAC), age, gender and receipt of other vaccines, all-cause gastroenteritis severity scores were 2.21 units lower (*p* < 0.001) among Rotarix^®^-vaccinated (*n* = 2224) compared to Rotarix^®^-unvaccinated children (*n* = 935). The reduction in severity score was observed against every rotavirus genotype, although the magnitude was smaller among those infected with G12P[6] compared to the remaining genotypes (*p* = 0.011). Each one-year increment in age was associated with a decrease of 0.43 severity score (*p* < 0.001). Our findings provide additional evidence on the impact of Rotarix^®^ in Malawi, lending further support to Malawi’s Rotarix^®^ programme.

## 1. Introduction

Rotavirus is a leading cause of acute gastroenteritis among children worldwide. Despite the introduction of rotavirus vaccines in many countries, rotavirus is still associated with an estimated 128,500 deaths annually. Over 90% of these cases occur in low- and middle-income countries (LMICs) in sub-Saharan Africa and South-East and South Asian countries [1,2].

Rotavirus has a segmented double-stranded ribonucleic acid (dsRNA) genome, surrounded by a triple-layered capsid. Most human infections are associated with group A rotaviruses [3], which can further be classified using a dual classification system into G and P types, according to their neutralising antibody response or nucleotide differences in the genes encoding their outer glycoprotein VP7 and protease-sensitive VP4, respectively [2]. At least 41 G types and 57 P types have been reported (https://rega.kuleuven.be/cev/viralmetagenomics/virus-classification, accessed on 12 December 2021). Of these, genotypes G1P[8], G2P[4], G3P[8], G4P[8], G9P[8] and G12P[8] are the most frequent causes of rotavirus disease in humans worldwide [4,5].

Malawi introduced the G1P[8] Rotarix^®^ rotavirus vaccine into its national Expanded Programme on Immunisation (EPI) schedule on 28 October 2012, with doses administered at 6 and 10 weeks of age. Rotarix^®^ vaccination coverage reached 99–100% by 2016 [6], and this was associated with a decline in rotavirus-associated hospitalisations [7] and a reduction in gastroenteritis-related mortality [8]. However, the impact of Rotarix^®^ vaccination on the severity of gastroenteritis presented in vaccinated and unvaccinated (indirect) children has not yet been assessed. We conducted an analysis of the severity of gastroenteritis by comparing Ruuska and Vesikari disease severity scores [9] in children presenting with rotavirus and non-rotavirus laboratory confirmed gastroenteritis before and after Rotarix^®^ introduction, and between vaccinated and non-vaccinated children post-Rotarix^®^ introduction.

## 2. Materials and Methods

### 2.1. Study Population

Children under the age of five years who presented with acute gastroenteritis (defined as the passage of at least three looser-than-normal stools in a 24 h period for less than seven days duration) whose mothers/legal guardians consented to participate in this study were enrolled at both inpatient and outpatient departments, Queen Elizabeth Central hospital (QECH), Blantyre, which is the main referral hospital for the southern region of Malawi. To assess the impact of Rotarix^®^ vaccination on the severity of gastroenteritis, data were examined from children requiring hospitalisation with gastroenteritis before (December 2011 to October 2012) and after (November 2012 to October 2019) Rotarix^®^ introduction. These populations were used to (i) assess the impact of Rotarix^®^ on the severity of all-cause gastroenteritis; and (ii) determine whether gastroenteritis severity differed by rotavirus genotype in Rotarix^®^-vaccinated and Rotarix^®^-unvaccinated children.

### 2.2. Clinical and Demographic Variables

Gastroenteritis severity was determined using the Ruuska and Vesikari scoring system [9]. The assessment was based on the following parameters: duration and maximum number of episodes of diarrhoea as well as vomiting, fever, and dehydration. Scores of 0–5 was considered as mild, 6–10 as moderate, 11–15 as severe and ≥16 as very severe. To assess the impact of Rotarix^®^ by age, infants were categorised into four age groups (<6, 6–11, 12–23 and 24–59 months).

### 2.3. Rotavirus Detection and Genotyping

A 10–20% stool suspension in diluent buffer was prepared for each specimen and used to screen for the presence of group A rotavirus using a commercially available enzyme immunoassay (Rotaclone^®^, Meridian Bioscience, Cincinnati, OH, USA). Rotavirus dsRNA was extracted from all rotavirus-positive stool samples using the Viral RNA Mini-Kit (Qiagen, Hilden, Germany). The dsRNA was reverse transcribed to complementary DNA (cDNA) using random primers (Invitrogen, Paisley, UK) and reverse transcriptase enzyme (Superscript III MMLV-RT, Invitrogen, Paisley, UK) [10]. The cDNA was used to assign G genotype (G1, G2, G3, G4, G8, G9, G10, G11 and G12) and P genotype (P[4], P[6], P[8], P[9], P[10], P[11] and P[14]) using a multiplex heminested RT-PCR as described previously [5].

### 2.4. Statistical Analysis

All statistical analyses were performed in the R environment for statistical computing, version 4.0.2 [11] and GraphPad Prism version 8. Vesikari score distributions were compared between pre-Rotarix^®^-unvaccinated children, post-Rotarix^®^-unvaccinated children and post-Rotarix^®^-vaccinated children as well as between genotypes using non-parametric Kruskal–Wallis tests. Wilcoxon rank-sum tests were used to compare Vesikari scores between two groups. A linear regression model was used to estimate the change in Vesikari score that could be associated with receipt of Rotarix^®^ and Rotarix^®^ vaccine period. This model was adjusted for the mid-upper arm circumference (MUAC), age, gender and receipt of the Bacillus Calmette–Guérin vaccine (BCG), the Pneumococcal Conjugate Vaccine (PCV), the Oral Polio Vaccine (OPV) and the Pentavalent (diphtheria, pertussis, tetanus, and hepatitis B and *Haemophilus influenzae* type b) EPI vaccines. This model was inspected for multicollinearity using generalised variable inflation factors (GVIFs) as implemented in the R package car [12]. No substantial multicollinearity was detected (all squared GVIFs < 5). Both unadjusted and adjusted linear regression analysis were used to estimate Vesikari scores by considering G genotypes separately, P genotypes separately and combined G and P genotypes (Supplementary Data S1). Residuals were computed for the purpose of model diagnostics: homoscedasticity and normality of residuals, linearity of the relationship between the independent and dependent variables.

## 3. Results

### 3.1. Reduction in Gastroenteritis Severity following Vaccination with Rotarix^®^

The characteristics of the study participants included in this analysis are summarised in Table 1. In total, 3159 children were enrolled, of which 401 (12.7%) were enrolled before Rotarix^®^ introduction, whereas 2758 (87.3%) were enrolled after Rotarix^®^ introduction. A total of 80.6% (2224/2758) of children enrolled in the post-Rotarix^®^ period were vaccinated. Thus, across the entire study period, 70.4% (2224/3159) of the children were vaccinated with Rotarix^®^. The median age of unvaccinated children was higher (15.9 (IQR: 9.1–19.9)) than that of vaccinated children (10.4 (IQR: 7.7–14.4)) during the post-vaccine period, *p* < 0.001.

**Figure 1:**
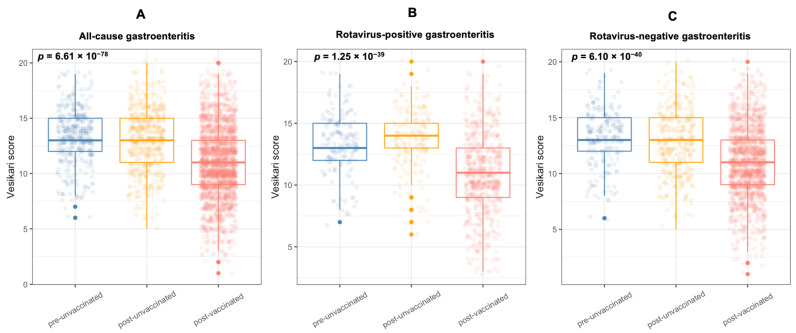
Ruuska and Vesikari severity scores among children hospitalised with gastroenteritis at QECH in Blantyre, Malawi before (December 2011 to October 2012) and after (November 2012 to October 2019) introduction of Rotarix^®^. (**A**) Severity scores in all-cause gastroenteritis cases. (**B**) Severity scores in rotavirus-positive cases. (**C**) Severity scores in rotavirus-negative cases. Testing the null hypothesis that the severity scores have the same distribution in all three groups was highly statistically significant (*p* = 6.61 × 10^−78^, 1.25 × 10^−39^, 6.10 × 10^−40^) for (**A**–**C**), respectively; Kruskal–Wallis test). The *p*-value for observing the data under the null hypothesis of no different distribution between groups was 0.260 using a Wilcoxon rank-sum test when only comparing the pre-Rotarix with the post-Rotarix^®^-unvaccinated group (for all gastroenteritis cases). Vesikari scores = Ruuska and Vesikari scores.

**Figure 2:**
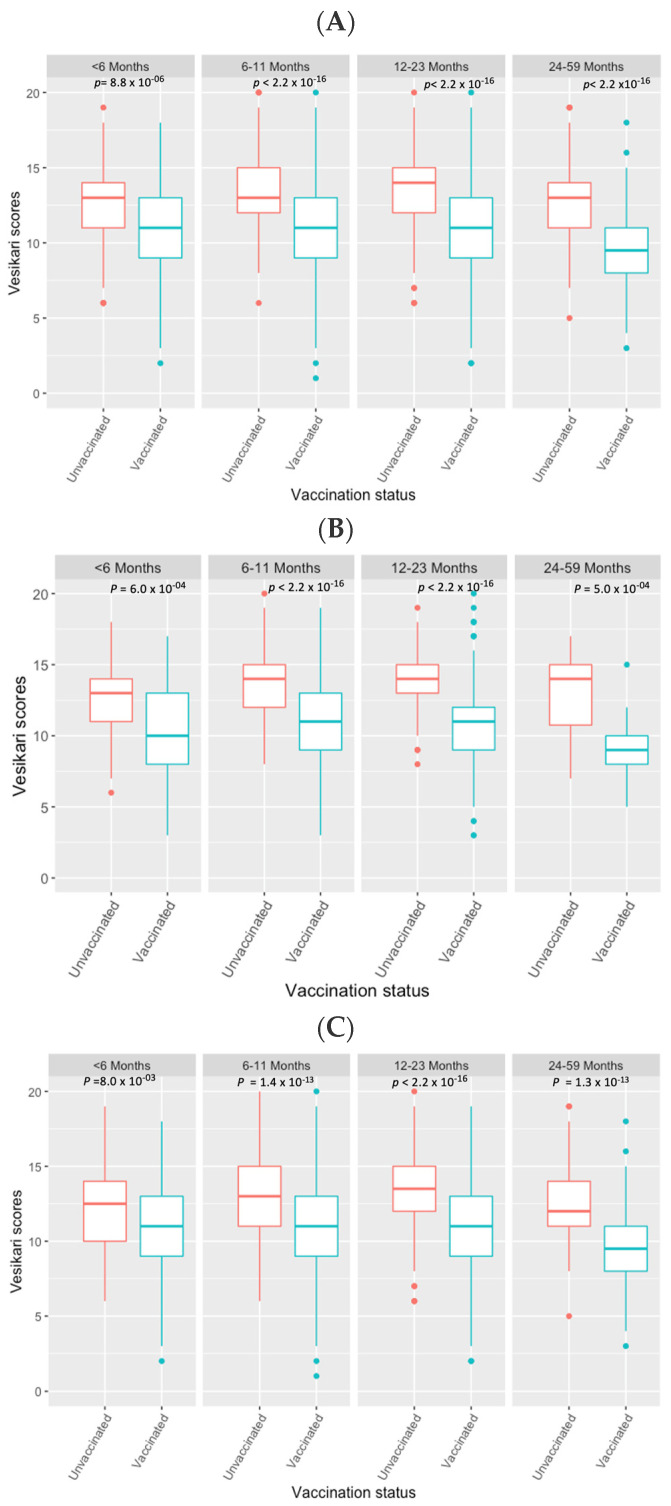
Comparison of Ruuska and Vesikari severity scores among children requiring hospitalisation with gastroenteritis at QECH in Blantyre, Malawi from December 2011 to October 2019 stratified by Rotarix^®^ vaccination status between different age groups (Kruskal–Wallis test). (**A**) Severity scores in all-cause gastroenteritis cases (Rotarix^®^-vaccinated, *n* = 248, 1128, 712 and 136; Rotarix^®^-unvaccinated, *n* = 121, 386, 311 and 117 in less than 6-, 6–11-, 12–23- and 24–59-month-old children, respectively). (**B**) Severity scores in rotavirus-positive cases (Rotarix^®^-vaccinated, *n* = 61, 353, 213, 25; Rotarix^®^-unvaccinated, *n* = 69, 156, 112 and 16 in less than 6-, 6–11-, 12–23- and 24–59-month-old children, respectively). (**C**) Severity scores in rotavirus-negative cases (Rotarix^®^-vaccinated, *n* = 187, 773, 498 and 110; Rotarix^®^-unvaccinated, *n* = 52, 225, 198 and 101 in less than 6-, 6–11-, 12–23- and 24–59-month-old children, respectively).

Rotarix^®^-vaccinated children presented with less severe gastroenteritis compared with Rotarix^®^-unvaccinated children during the post-vaccine period (unadjusted Kruskal–Wallis test, *p* < 0.001). There was no difference in the severity of all-cause gastroenteritis between Rotarix^®^-unvaccinated children before and after Rotarix^®^ introduction (unadjusted Wilcoxon rank-sum test, *p* = 0.260) (Figure 1a). When Rotarix^®^-vaccinated children were stratified into rotavirus-positive and rotavirus-negative cases, a decrease in severity score was observed in Rotarix^®^-vaccinated children for both groups (Figure 1b,c). Reductions in all-cause gastroenteritis severity three years or later following Rotarix^®^ introduction were observed in all age groups (Figure 2, Figure S1 and Table S1).

Unadjusted regression analysis confirmed a linear relationship between the reduction in all-cause gastroenteritis severity and vaccination with Rotarix^®^ when pre-Rotarix^®^-unvaccinated gastroenteritis cases requiring hospitalisation were used as a reference group. There was an average estimated reduction of 2.35 (95% confidence interval (CI) 2.03, 2.67; *p* < 0.001) in severity scores among Rotarix^®^-vaccinated children and no reduction among Rotarix^®^-unvaccinated children during the post-vaccine period (0.22; 95% CI −0.16, 0.61; *p* = 0.260). Adjusting for the MUAC, age, gender, and EPI vaccination status in the linear regression did not substantially change the estimated reduction in severity scores among Rotarix^®^-vaccinated (2.21; 95% CI: 1.85, 2.56; *p* < 0.001) and Rotarix^®^-unvaccinated children (0.05; 95% CI −0.46, 0.36; *p* = 0.820] (Table S2). Unlike the other covariates, there was some evidence of a linear association between age and gastroenteritis severity when children enrolled before Rotarix^®^ introduction and without a Rotarix^®^ vaccination history were used as a reference group: every increment of 1 year in the age was associated with a decrease of 0.43 (95% CI 0.26, 0.60; *p* < 0.001) in Ruuska and Vesikari scores (Table S2). Except for <6-month-old rotavirus-positive children where severity started to decline from the 2013–2014 calendar year, at least a year post-Rotarix^®^ introduction, substantial decline in severity was observed in all age groups between 2014 and 2015, at least three years after Rotarix^®^ introduction (Figure S1). Severity scores increased in all cases from 2017 to 2019 (Figure S1).

### 3.2. Relationship between Rotavirus Genotype, Gastroenteritis Severity and Vaccination with Rotarix^®^

**Figure 3:**
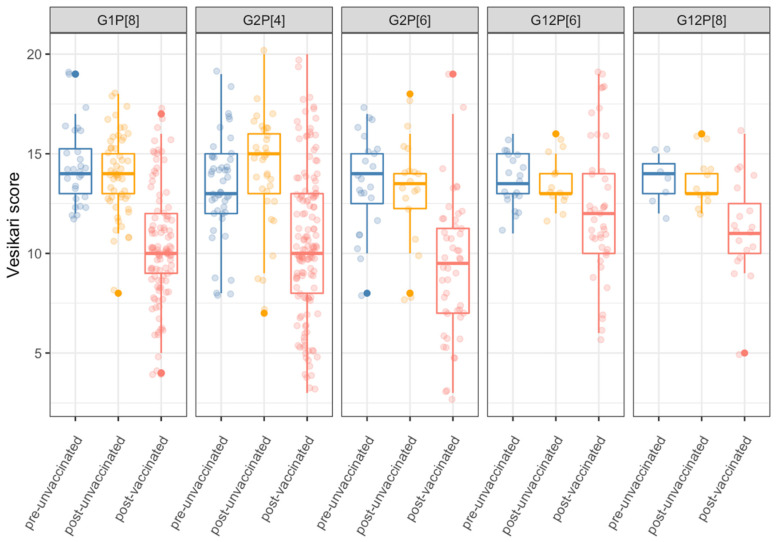
Comparison of severity scores in Rotarix^®^-vaccinated and Rotarix^®^-unvaccinated hospitalised children at Queen Elizabeth Central Hospital in Blantyre, Malawi presenting with gastroenteritis stratified by frequently detected combined G and P rotavirus genotypes. Vesikari scores = Ruuska and Vesikari scores.

The most frequently detected rotavirus genotypes were G1P[8], G2P[4], G2P[6], G12P[6] and G12P[8] [5], which comprised 66.57% (636/1050) of all genotypes (Table S3). In Rotarix^®^-unvaccinated children, regardless of the genotype, most gastroenteritis episodes were classified as severe (66.67%, 424/636) and severity scores did not differ by genotype (unadjusted Kruskal–Wallis test, *p* = 0.544). In Rotarix^®^-vaccinated children, a decrease in severity was observed for infections with all rotavirus genotypes compared to Rotarix^®^-unvaccinated children, with most vaccinated children having moderate disease. This decrease in severity was less pronounced in cases infected with G12P[6] and G12P[8] genotypes; and Rotarix^®^-vaccinated children infected with G12P[6] strains had a 2.58 unit (95% CI 0.60, 4.56; *p* = 0.011) higher severity score when compared with the remaining strains after adjusting for age (Figure 3 and Supplementary Table S4). Stratifying severity scores by G and P genotypes separately suggested that this effect might be associated with the G12 genotype (Figure S2). There were no differences in severity scores between pre-Rotarix^®^ and post-Rotarix^®^-unvaccinated groups in G and P genotypes, but a clear difference within the post-Rotarix^®^-vaccinated group (Figure S2). The decrease in severity among post-Rotarix^®^-vaccinated children was more pronounced for genotypes G1 and G2 compared to G12 (unadjusted Kruskal–Wallis test, *p* < 0.001). Adjusting for age did not affect the regression outcomes (data not shown).

## 4. Discussion

Introduction of Rotarix^®^ rotavirus vaccine into Malawi’s childhood immunisation schedule was associated with a significant reduction in the severity of all-cause gastroenteritis presented by children requiring hospitalisation under the age of five years at QECH in Blantyre. Irrespective of the rotavirus genotype, Rotarix^®^-vaccinated children presented with less severe rotavirus disease compared to Rotarix^®^-unvaccinated children. The reduction in gastroenteritis severity was less pronounced in vaccinated children infected with G12 rotaviruses compared to other common genotypes such as G1 and G2 that circulated in Blantyre before and after introduction of Rotarix^®^ vaccine in Malawi.

Rotarix^®^ was developed to prevent children from developing severe gastroenteritis following infection with rotaviruses post-vaccination and not necessarily to prevent them from getting infected with rotaviruses [13,14]. Thus, our study demonstrates the expected, but to date unmeasured, direct impact of Rotarix^®^ vaccine in reducing the severity of disease in children with acute rotavirus gastroenteritis in a low-income setting. Rotarix^®^ is administered at 6th and 10th week of age in Malawi, hence children older than 2.5 months were ineligible to receive Rotarix^®^ when it was introduced in October 2012. Thus, the median age for the Rotarix^®^-unvaccinated children enrolled into our active diarrhoea surveillance platform at QECH was significantly higher compared to Rotarix^®^-vaccinated children. Although rotavirus-associated severe diarrhoea cases are more common in younger children [1,15], we observed that Rotarix^®^-unvaccinated children, who were much older, presented with more severe diarrhoea compared to Rotarix^®^-vaccinated ones who were younger in Malawi. The majority of the older Rotarix^®^-unvaccinated children presented with rotavirus-negative diarrhoea, supporting what is known that rotavirus infection in common in younger children [1,15,16].

Age-associated reductions in the probability for primary, secondary, and subsequent rotavirus infections to cause rotavirus gastroenteritis have been demonstrated previously [17]. Human studies on factors underlying age-related determinants of risks to rotavirus gastroenteritis are not available but studies in animal models suggest that immune maturation, postnatal intestinal development, and establishment of the gut microbial communities are some of the factors that contributes to age-dependent risk for infection to cause rotavirus gastroenteritis [18,19,20,21]. These observations could explain the reduction in the severity of gastroenteritis in children >23 months regardless of their Rotarix^®^-vaccination status observed in our study which is consistent with findings from elsewhere [17,22,23]. In contrast, the severity scores were similar between different age groups of either Rotavirus^®^-vaccinated or Rotavirus^®^-unvaccinated children <23 months that we analysed but were significantly different between Rotavirus^®^-vaccinated and Rotavirus^®^-unvaccinated children in all age strata—this difference in severity could be attributed to rotavirus vaccination.

We also identified unexpected off-target vaccine benefit as Rotarix^®^ recipients presented with less severe gastroenteritis regardless of the presence or absence of rotavirus. This is consistent with previous findings in Malawi and other settings where introduction of Rotarix^®^ was associated with a reduction in all-cause diarrhoea mortality [8,24,25,26,27]. Nonspecific vaccine effects were previously observed in infants vaccinated with BCG [28,29], measles [30] and other live attenuated vaccines such as the trivalent oral polio vaccine (OPV) [26,31]. T cell-mediated cross-reactivity and trained innate immunity are among the mechanisms that may explain the off-target vaccine benefit [32,33]. Vaccination with BCG elevates innate immune markers, such as IFN-γ, tumour necrosis factor α, interleukin 1βeta (IL-1β) and IL-6 cytokines [34,35]. Oral live attenuated rotavirus vaccines could potentially employ similar mechanisms as they have been shown to induce innate immune responses [36] and effectively replicate in the gut of vaccinated children, [37,38,39] which could trigger cross-reactive CD8+ T-cell responses. In addition, it is possible that averting rotavirus diarrhoea or experiencing less severe gastroenteritis in Rotarix^®^-vaccinated children [40,41] promotes a healthier gut compared to their Rotarix^®^-unvaccinated counterparts. The impaired gut integrity and physiology that follows severe rotavirus infection, may render them more susceptible to infections by other enteropathogens. This may potentially contribute, in part, to the indirect effects of rotavirus vaccine previously reported as herd protection which is thought to be caused by decreased force of infection due to increased prevention of a disease targeted by a specific vaccine [24,25,42,43,44].

Rotarix^®^ vaccination was associated with a reduction in rotavirus gastroenteritis severity regardless of the infecting rotavirus genotype. Although Rotarix^®^ effectiveness was previously documented to be lower against genotype G2-associated gastroenteritis compared with disease associated with genotype G1 [5,45,46], the reduction in severity in cases associated with G1 and G2 genotypes was similar among Rotarix^®^-vaccinated children in the current study. However, the decline in gastroenteritis severity was less pronounced among cases associated with the G12 genotype. While the reasons for this observation are unknown, a relatively lower Rotarix^®^ effectiveness has been reported against some heterotypic rotavirus strains, including G12s in this population [45]. Future genomic and immunological studies are warranted to investigate how neutralising antibodies induced by a G1P[8] Rotarix^®^ vaccine would effectively binds to antigenic regions of various heterotypic rotavirus strains owing to the vast amount of amino acid differences that have been observed between rotaviruses bearing different VP4 and VP7 genotypes [2,47,48]. Intriguingly, from 2017, rotavirus and non-rotavirus gastroenteritis severity scores increased in all age groups, although they remained lower than among those in unvaccinated children (Figure S1). Such a trend may be explained by potential changes in the criteria for hospitalisation of children presenting with gastroenteritis at QECH or changes in health seeking behaviour and or access of the communities around Blantyre post-2017. Further investigations are warranted to understand the significance and causes of this trend.

We could not test the trend in reduction of severity between vaccinated and unvaccinated infants because the number of unvaccinated infants decreased during each consecutive year due to increase in Rotarix^®^ vaccine coverage. In addition, our analysis was limited by the availability of gastroenteritis cases from only one surveillance year prior to Rotarix^®^ introduction. A further limitation is that all children required hospitalisation, and hence had at least moderately severe gastroenteritis. Future studies should also examine the benefits of rotavirus vaccination in children with less severe gastroenteritis treated as outpatients at surrounding health care facilities. Finally, the observed non-specific reduction in all-cause gastroenteritis could not be attributed to individual diarrhoea pathogens as the stool specimens were not routinely screened for enteric pathogens beyond rotavirus.

## 5. Conclusions

Our study provides evidence of a reduction in gastroenteritis severity among hospitalised children who had been vaccinated with Rotarix^®^ vaccine seven years following its introduction in Malawi’s immunization programme. Rotavirus vaccination reduced the severity of rotavirus gastroenteritis caused by both homotypic and heterotypic rotavirus strains. Furthermore, rotavirus vaccination decreased non-rotavirus gastroenteritis severity, suggesting important off-target vaccine effects. Overall, these data demonstrate previously unmeasured direct and indirect benefits of rotavirus vaccines in Malawian children, providing further support for their continued programmatic use.

## Figures and Tables

**Table 1:** Characteristics of children who presented with gastroenteritis at the Queen Elizabeth Central Hospital in Blantyre, Malawi from December 2011 to October 2019.

		All-Cause Gastroenteritis			Rotavirus-Positive Gastroenteritis			Rotavirus-Negative Gastroenteritis		
		Pre-Rotarix^®^ Introduction Period	Post-Rotarix^®^ Introduction Period		Pre-Rotarix^®^ Introduction Period	Post-Rotarix^®^ Introduction Period		Pre-Rotarix^®^ Introduction Period	Post-Rotarix^®^ Introduction Period	
Variable			Rotarix^®^-Vaccinated	Rotarix^®^-Unvaccinated		Rotarix^®^-Vaccinated	Rotarix^®^-Unvaccinated		Rotarix^®^-Vaccinated	Rotarix^®^-Unvaccinated
Total participants; *n* (%)		401 (12.7%)	2224 (70.4%)	534 (16.9%)	176 (17.5%)	652 (64.9%)	177 (17.6%)	225 (10.4%)	1572 (73.0%)	357 (16.6%)
Sex; *n* (%)	Male	218 (54.4%)	1354 (60.9%)	301 (56.4%)	95 (54.0%)	401 (61.5%)	97 (54.8%)	123 (54.7%)	953 (60.6%)	204 (57.1%)
	Female	183 (45.6%)	870 (39.1%)	233 (43.6%)	81 (46.0%)	251 (38.5%)	80 (45.2%)	102 (45.3%)	619 (39.4%)	153 (42.9%)
Year of surveillance; *n* (%)	2011–2012	401 (100%)	0 (0%)	0 (0%)	176 (100%)	NA	NA	225 (100%)	0 (0%)	0 (0%)
	2012–2013	NA	162 (29.2%)	393 (70.8%)	NA	54 (27.3%)	144 (72.7%)	NA	108 (30.3%)	249 (69.7%)
	2013–2014	NA	450 (82.4%)	96 (17.6%)	NA	108 (85.0%)	19 (15.0%)	NA	342 (81.6%)	77 (18.4%)
	2014–2015	NA	513 (94.6%)	29 (5.4%)	NA	126 (94.0%)	8 (6.0%)	NA	387 (94.9%)	21 (5.1%)
	2015–2016	NA	364 (96.6%)	13 (3.4%)	NA	116 (95.1%)	6 (4.9%)	NA	248 (97.3%)	7 (2.7%)
	2016–2017	NA	313 (96.9%)	10 (3.1%)	NA	106 (96.4%)	4 (3.6%)	NA	207 (97.2%)	6 (2.8%)
	2017–2018	NA	213 (99.5%)	1 (0.5%)	NA	69 (100%)	0 (0%)	NA	144 (99.3%)	1 (0.7%)
	2018–2019	NA	200 (99.5%)	1 (0.5%)	NA	69 (100%)	0 (0%)	NA	131 (99.2%)	1 (0.8%)
Vesikari score; median (IQR)		13.0 (12.0–15.0)	11.0 (9.0–13.0)	13.0 (11.0–15.0)	13.0 (12.0–15.0)	11.0 (9.0–14.0)	14.0 (13.0–15.0)	13.0 (12.0–15.0)	11.0 (9.0–13.0)	13.0 (11.0–15.0)
MUAC (cm); median (IQR)		13.0 (12.0–14.0)	13.1 (12.5–14.0)	13.5 (12.50–14.0)	13.0 (12.0–14.0)	13.2 (12.5–14.0)	13.4 (12.5–14.0)	13.0 (11.6–14.0)	13.1 (12.4–14.0)	13.5 (12.5–14.3)
Weight (kg); median (IQR)		7.6 (6.4–9.0)	7.9 (6.9–9.0)	8.0 (6.8–9.7)	7.5 (6.4–8.5)	7.9 (7.0–9.0)	8.0 (6.8–9.2)	7.8 (6.5–9.3)	7.9 (6.80–9.0)	8.0 (6.8–9.9)
Age (months); median (IQR)		9.5 (6.9–13.4)	10.4 (7.7–14.4)	15.9 (9.1–19.9)	8.1 (5.8–11.1)	10.3 (7.9–13.8)	12.1 (8.3–15.7)	10.6 (7.7–15.5)	10.4 (7.7–14.7)	14.6 (9.5–22.0)
BCG-Vaccinated; *n* (%)	Yes	381 (95.0%)	2199 (98.9%)	519 (97.2%)	165 (93.8%)	645 (98.9%)	171 (96.6%)	216 (96.0%)	1554 (98.9%)	348 (97.5%)
	No	20 (5%)	25 (1.1%)	15 (2.8%)	11 (6.3%)	7 (1.1%)	6 (3.4%)	9 (1.1%)	18 (1.1%)	9 (2.5%)
Pentavalent-vaccinated; *n* (%)	Yes	392 (97.8%)	2219 (99.8%)	507 (94.9%)	171 (97.2%)	651 (99.8%)	165 (93.4%)	221 (99.7%)	1568 (99.7%)	342 (95.8%)
	No	9 (2.2%)	5 (0.2%)	27 (5.1%)	5 (2.8%	1 (0.2%)	12 (6.8%)	4 (0.4%)	4 (0.3%)	15 (4.2%)

MUAC; mid-upper arm circumference. BCG; Bacillus Calmette–Guérin (BCG) vaccine. Pentavalent vaccine containing five antigens (diphtheria, pertussis, tetanus, and hepatitis B and Haemophilus influenzae type b). NA = not applicable. Vesikari scores = Ruuska and Vesikari scores.

## Data Availability

The data presented in this study are available on request from the corresponding author. The data are not publicly available due to ethical restrictions.

## Supplementary Materials

The following are available online at https://www.mdpi.com/article/10.3390/v13122491/s1, Supplementary Data S1. Unadjusted and adjusted linear regression models used to estimate the change in Vesikari score; Figure S1. Trends in gastroenteritis severity among all children who presented with gastroenteritis at Queen Elizabeth Central Hospital in Blantyre, Malawi during the pre- (December 2011 to October 2012) and post-Rotarix^®^ (November 2012 to October 2019) periods amongst different age groups; Figure S2. Severity scores in hospitalised children with rotavirus confirmed gastroenteritis at Queen Elizabeth Central Hospital, Blantyre, Malawi; Table S1. Gastroenteritis severity based on Ruuska and Vesikari scores among children presenting with gastroenteritis at the Queen Elizabeth Central Hospital in Blantyre, Malawi between the pre- (December 2011–October 2012) and post-vaccine (November 2012–October 2019) introduction periods; Table S2. Unadjusted and adjusted linear regression model for the estimated reduction in severity scores among hospitalised children at Queen Elizabeth Central Hospital in Blantyre, Malawi after Rotarix^®^ introduction; Table S3. Frequently detected rotavirus strains and their gastroenteritis severity scores in children presenting with gastroenteritis at Queen Elizabeth Central Hospital, Blantyre, Malawi during the pre- and post-Rotarix^®^ period (December 2011 to October 2019); Table S4. Linear regression model for estimated reduction in severity scores and genotype (G and P), adjusted for age, MUAC and EPI vaccination status.

## Disclaimer

The funders had no role in the study design, data collection and interpretation, or the decision to submit the work for publication. The authors did not receive any financial support or other form of reward related to the development of the manuscript. Therefore, findings and conclusions in this report are those of the authors and do not necessarily represent the formal position of the funders. D.H, M.IG., NAC. and K.C.J are affiliated to the National Institute for Health Research (NIHR) Health Protection Research Unit in Gastrointestinal Infections at University of Liverpool, a partnership with Public Health England, in collaboration with University of Warwick. The views expressed are those of the author(s) and not necessarily those of the NIHR, the Department of Health and Social Care or Public Health England.
