# Supplementary material for: Reduction in Severity of All-Cause Gastroenteritis Requiring Hospitalisation in Children Vaccinated against Rotavirus in Malawi
Source: Viruses. 2021 Dec 13;13(12):2491. doi: 10.3390/v13122491 (PMC8707889; doi:10.3390/v13122491)
Supplement: Supplementary file 1 [file viruses-13-02491-s001.zip › Supplementary Data S1_Viruses_Submission_Proofed.pdf]

Supplementary Data S1. Unadjusted and adjusted linear regression models used to estimate the change in Vesikari score

A linear regression model (*Vesikari score* = $\beta_0 + \beta_1 *$ [post Rotarix®-unvaccinated] + $\beta_2 *$ [post RV2-vaccinated] + ϵ) was used to estimate the change in Vesikari score that could be associated with receipt of Rotarix® and Rotarix® vaccine period. This model was adjusted for the Mid-Upper Arm Circumference (MUAC), age, weight, gender and receipt of the Bacillus Calmette–Guérin (BCG), Pneumococcal Conjugate Vaccine (PCV), Oral Polio Vaccine (OPV) and Pentavalent (diphtheria, pertussis, tetanus, and hepatitis B and *Haemophilus influenzae* type b) EPI vaccines. Both unadjusted and adjusted linear regression analysis were used to estimate Vesikari scores by considering G genotypes separately, P genotypes separately and combined G and P genotypes (Supplementally Data 1). For instance, the following unadjusted model was used to estimate effects of combined G & P genotypes: Vesikari = $\beta_0 + \beta_1 *$ [post Rotarix®-unvaccinated] + $\beta_2 *$ [post RV2-vaccinated] + β_3 [genotype G2P[4]] +β_4 [genotype G2P[6]] + β_5 [genotype G12P[6]] +β_6 [genotype G12P[8]] + β_7 [post Rotarix®-unvaccinated * genotype G2P[4]] + β_8 [post Rotarix®-unvaccinated * genotype G2P[6]] + β9 [post Rotarix®-unvaccinated * genotype G12P[6]] + β_{10} [post Rotarix®-unvaccinated * genotype G12P[8]] + β11 [post Rotarix®-vaccinated * genotype G2P[4]] + β12 [post Rotarix®-vaccinated *genotype G2P[6]]+ β_{13} [post Rotarix®-vaccinated * genotype G12P[6]] + β_{14} [post Rotarix®-vaccinated * genotype G12P[8]] + ϵ. Residuals were computed for the purpose of model diagnostics: homoscedasticity and normality of residuals, linearity of the relationship between the independent and dependent variables.
