# Supplementary material for: Reduction in Severity of All-Cause Gastroenteritis Requiring Hospitalisation in Children Vaccinated against Rotavirus in Malawi
Source: Viruses. 2021 Dec 13;13(12):2491. doi: 10.3390/v13122491 (PMC8707889; doi:10.3390/v13122491)
Supplement: Supplementary file 1 [file viruses-13-02491-s001.zip › Supplementary Data S2_Table_S1_Viruses_Submission_Profeed.pdf]

Supplementary Data S2, Table S1. Gastroenteritis severity based on Ruuska and Vesikari scores among children presenting with gastroenteritis at the Queen Elizabeth Central Hospital in Blantyre, Malawi between the pre- (December 2011 – October 2012) and post-vaccine (November 2012 – October 2019) introduction periods.

	Vaccination period						Comparison between vaccination periods		
	Pre-Rotarix®[1]		Post-Rotarix® Unvaccinated[2]		Post-Rotarix® Vaccinated[3]		Pre-Rotarix® vs Post-Rotarix® Unvaccinated	Pre-Rotarix® vs Post-Rotarix® Vaccinated	Post-Rotarix® Vaccinated vs Post-Rotarix® Unvaccinated
**Age (Mons[4])**	Number of samples	Vesikari scores($\bar{x}$)	Number of samples	Vesikari scores($\bar{x}$)	Number of samples	Vesikari scores($\bar{x}$)	*p*-values		
	**All gastroenteritis cases[5]**								
**<6**	72	12.2	49	12.6	248	10.7	0.488	< 0.001	0.058
**6 – 11**	203	13.5	183	13.2	1128	11.3	0.228	< 0.001	< 0.001
**12 – 23**	94	13.8	217	13.5	712	10.8	0.19	< 0.001	< 0.001
**24 – 59**	32	13.2	85	12.3	136	9.5	0.084	< 0.001	< 0.001
**0 – 59**	401	13.3	534	13	2224	11	0.194	< 0.001	< 0.001
	**Rotavirus positive gastroenteritis cases[6]**								
**<6**	45	12.3	24	12.6	61	10.4	0.36	0.001	0.17
**6 – 11**	94	13.7	62	13.9	353	11.1	0.352	< 0.001	< 0.001
**12 – 23**	35	14.2	77	13.9	213	10.7	0.364	< 0.001	< 0.001
**24 – 59**	2	13.5	16	12.8	24	9.2	0.496	0.006	< 0.001
**0 – 59**	176	13.4	177	13.6	652	10.9	0.421	< 0.001	< 0.001
	**Rotavirus negative gastroenteritis cases[7]**								
**<6**	27	12	25	126	187	10.8	0.549	0.044	0.156
**6 – 11**	104	13.4	121	12.9	773	11.4	0.128	< 0.001	< 0.001
**12 – 23**	58	13.6	140	13.2	498	10.9	0.269	< 0.001	< 0.001
**24 – 59**	30	13.2	71	12.2	110	9.6	0.045	< 0.001	< 0.001
**0 – 59**	219	13.3	357	12.8	1568	11	0.046	< 0.001	< 0.001

[1]Before Rotarix® was introduced. [2] Rotarix®-unvaccinated children during the Rotarix® period. [3] Rotarix®-vaccinated children during the Rotarix® period. [4]Age in months. [5]Compares severity of gastroenteritis between vaccination periods and vaccination status amongst different age groups for all gastroenteritis cases. [6]Compares severity of gastroenteritis between vaccination periods and vaccination status amongst different age groups for rotavirus-related gastroenteritis cases.[7]Compares severity of gastroenteritis between vaccination periods and vaccination status amongst different age groups for non-rotavirus related gastroenteritis cases. Vesikari scores = Ruuska and Vesikari scores.
