# Supplementary material for: Reduction in Severity of All-Cause Gastroenteritis Requiring Hospitalisation in Children Vaccinated against Rotavirus in Malawi
Source: Viruses. 2021 Dec 13;13(12):2491. doi: 10.3390/v13122491 (PMC8707889; doi:10.3390/v13122491)
Supplement: Supplementary file 1 [file viruses-13-02491-s001.zip › Supplementary Data S3_Table_S2_Viruses_Submission_Proofed.pdf]

Supplementary Data S3, Table S2. Unadjusted and adjusted linear regression model for the estimated reduction in severity scores among hospitalised children at Queen Elizabeth Central Hospital in Blantyre, Malawi after Rotarix® introduction.

Variable	Coefficient estimate	95% confidence interval	*p*-value
	**Unadjusted [a]**		
Intercept	13.34	13.04 – 13.63	<0.001
Post- Rotarix® unvaccinated	-0.22	-0.61 – 0.16	0.260
Post- Rotarix® vaccinated	-2.351	-2.67 – -2.03	0.001
	**Adjusted [b]**		
Intercept	13.08	11.92 – 14.24	<0.001
Post- Rotarix® unvaccinated	0.05	-0.36 – 0.46	0.821
Post- Rotarix® vaccinated	-2.21	-2.56 – 1.85	<0.001
Age	-0.04	-0.05 – -0.02	<0.001
MUAC	0.01	-0.02 – 0.04	0.569
BCG	-0.35	-1.13 – 0.44	0.391
Penta	0.40	-1.22 – 2.02	0.630
Polio	0.92	-0.61 – 2.45	0.239
PCV	-0.55	-1.11 – 0.01	0.053

[a] Unadjusted for other covariates. Pre-Rotarix® is taken as the reference group. Adjusted R^2 = 0.10. [b] Adjusted for age, MUAC and vaccination status (BCG, penta, polio, PCV). Participants from the pre-Rotarix® period without any vaccinations were taken as the reference group. Adjusted R^2 = 0.11. MUAC; Middle-Upper Arm Circumference. BCG; Bacillus Calmette–Guérin (BCG) vaccine. PCV; Pneumococcal conjugate vaccine. Penta; Pentavalent vaccine containing five antigens (diphtheria, pertussis, tetanus, and hepatitis B and Haemophilus influenzae type b).
