# Supplementary material for: Reduction in Severity of All-Cause Gastroenteritis Requiring Hospitalisation in Children Vaccinated against Rotavirus in Malawi
Source: Viruses. 2021 Dec 13;13(12):2491. doi: 10.3390/v13122491 (PMC8707889; doi:10.3390/v13122491)
Supplement: Supplementary file 1 [file viruses-13-02491-s001.zip › Supplementary_Data S4_Figure_S1_Viruses_Submission_Proofed.pdf]

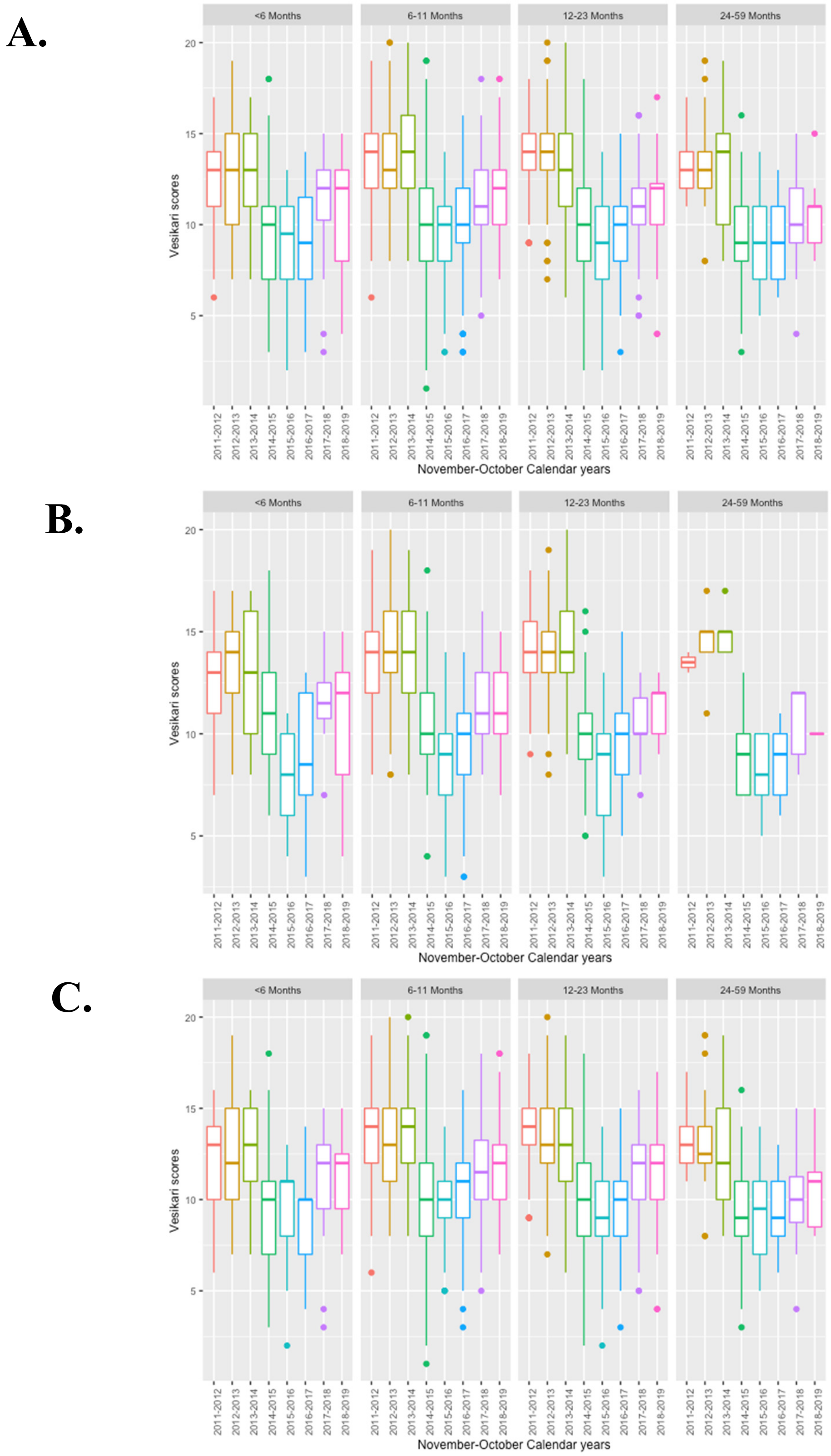

Supplementary Data S4, Figure S1. Trends in gastroenteritis severity among all children who presented with gastroenteritis at Queen Elizabeth Central Hospital in Blantyre, Malawi during the pre- (December 2011 to October 2012) and post-Rotarix® (November 2012 to October 2019) periods amongst different age groups. (A) All-cause gastroenteritis. (B) Rotavirus-positive gastroenteritis. (C) Rotavirus-negative gastroenteritis.
