# Supplementary material for: Reduction in Severity of All-Cause Gastroenteritis Requiring Hospitalisation in Children Vaccinated against Rotavirus in Malawi
Source: Viruses. 2021 Dec 13;13(12):2491. doi: 10.3390/v13122491 (PMC8707889; doi:10.3390/v13122491)
Supplement: Supplementary file 1 [file viruses-13-02491-s001.zip › Supplementary_Data S5_Table_S3_Viruses_Submission_Proofed.pdf]

Supplementary Data S5, Table S3. Frequently detected rotavirus strains and their gastroenteritis severity scores in children presenting with gastroenteritis at Queen Elizabeth Central Hospital, Blantyre, Malawi during the pre- and post-Rotarix® period (December 2011 to October 2019).

Gastroenteritis severity associated with frequently detected rotavirus genotypes										
	Mild		Moderate		Severe		Very severe		Total	
Genotypes	n	%	n	%	n	%	n	%	n	%
VP7 (G) Genotypes only[1]										
**G1**	4	1.6%	93	36.0%	129	50.0%	32	12.4%	258	100.0%
**G2**	28	7.7%	120	33.1%	165	45.5%	50	13.8%	363	100.0%
**G12**	1	0.7%	28	20.7%	91	67.4%	15	11.1%	135	100.0%
**Total**	**33**	**4.57%**	**241**	**31.88%**	**385**	**50.93%%**	**97**	**12.83%%**	**756**	**100.00%**
VP4 (P) Genotypes only[2]										
**P[4]**	17	5.3%	103	32.1%	155	48.3%	46	14.3%	321	100.0%
**P[6]**	7	3.0%	67	28.8%	134	57.5%	25	10.7%	233	100.0%
**P[8]**	9	3.0%	95	32.0%	162	54.5%	31	10.4%	297	100.0%
**Total**	**33**	**3.88%**	**265**	**31.14%%**	**451**	**53.00%**	**102**	**11.99%**	**851**	**100.00%**
Combined G and P Genotypes[3]										
**G1P[8]**	4	2.0%	63	31.8%	103	52.0%	28	14.1%	198	100.0%
**G2P[4]**	17	7.5%	69	30.5%	104	46.0%	36	15.9%	226	100.0%
**G2P[6]**	7	7.4%	31	32.6%	48	50.5%	9	9.5%	95	100.0%
**G12P[8]**	1	2.6%	7	18.4%	27	71.1%	3	7.9%	38	100.0%
**G12P[6]**	0	0.0%	13	16.5%	54	68.4%	12	15.2%	79	100.0%
**Total**	**29**	**4.46%**	**183**	**28.77%**	**336**	**52.83%**	**88**	**13.84%**	**636**	**100.00%**

[1] Numbers and proportions of four levels of gastroenteritis severity (Ruuska and Vesikari scores) for VP7 (G) genotypes only.
[2] Numbers and proportions of four levels of gastroenteritis severity (Ruuska and Vesikari scores) for VP4 (P) genotypes only.
[3] Numbers and proportions of four levels of gastroenteritis severity (Ruuska and Vesikari scores) for combined G and P genotypes.
