# Supplementary material for: Reduction in Severity of All-Cause Gastroenteritis Requiring Hospitalisation in Children Vaccinated against Rotavirus in Malawi
Source: Viruses. 2021 Dec 13;13(12):2491. doi: 10.3390/v13122491 (PMC8707889; doi:10.3390/v13122491)
Supplement: Supplementary file 1 [file viruses-13-02491-s001.zip › Supplementary_Data S6_Table_S4_Viruses_Submission_Proofed.pdf]

Supplementary Data S6, Table S4. Linear regression model for estimated reduction in severity scores and genotype (G and P), adjusted for age, MUAC and EPI vaccination status. Participants from the pre-Rotarix® period, with genotype G1P[8] and no vaccinations are taken as the reference group. Adjusted $R^2 = 0.24$.

**Variable**	**Coefficient Estimate**	**95% Confidence Interval**	***p*-value**
Intercept	14.53	11.77 – 17.29	<0.001
post-Rotarix® unvaccinated	-0.01	-1.40 – 1.39	0.992
post-Rotarix® vaccinated	-3.74	-5.01 – -2.47	<0.001
Genotype G2P[4]	-0.99	-2.37 – 0.39	0.159
Genotype G2P[6]	-0.64	-2.30 – 1.02	0.451
Genotype G12P[6] [1]	-0.64	-2.34 – 1.05	0.459
Genotype G12P[8]	-0.37	-2.82 – 2.07	0.765
Age	-0.03	-0.07 – 0.01	0.114
MUAC	0.02	-0.02 – 0.06	0.361
BCG	-0.37	-1.97 – 1.24	0.654
Penta	-0.66	-3.87 – 2.56	0.690
Polio	1.50	-0.95 – 3.95	0.232
PCV	-0.82	-1.98 – 0.34	0.169
Post-Rotarix® unvaccinated: genotype G2P[4]	1.20	-0.68 – 3.07	0.212
Post-Rotarix® vaccinated: genotype G2P[4]	1.26	-0.31 – 2.83	0.115
Post-Rotarix® unvaccinated: genotype G2P[6]	-0.61	-2.91 – 1.68	0.600
Post-Rotarix® vaccinated: genotype G2P[6]	-0.53	-2.47 – 1.41	0.591
Post-Rotarix® unvaccinated: genotype G12P[6]	0.06	-2.40 – 2.53	0.960
Post-Rotarix® vaccinated: genotype G12P[6]	2.58	0.60 – 4.56	0.011
Post-Rotarix® unvaccinated: genotype G12P[8]	-0.03	-3.12 – 3.06	0.983
Post-Rotarix® vaccinated: genotype G12P[8]	1.29	-1.54 – 4.12	0.373

[1] Rotarix®-vaccinated children had severity scores on average 3.74 (95% CI [2.47, 5.01]; p<0.0001) units lower than Rotarix®-unvaccinated children, and while G12P[6] infections had on average severity scores 0.64 (95% CI [-1.05, 2.34]; p=0.4589) units higher compared to G1P[8] genotypes, this combined effect (average decrease of 3.74 – 0.64 = 3.10) was offset on average by an increase of 2.58 (95% CI [0.60, 4.56]; p=0.0111) units for children which were both Rotarix®-vaccinated and G12P[6] infected. MUAC; Middle-Upper Arm Circumference. BCG; Bacillus Calmette–Guérin (BCG) vaccine. PCV; Pneumococcal conjugate vaccine. Penta: Pentavalent vaccine containing five antigens (diphtheria, pertussis, tetanus, and hepatitis B and Haemophilus influenzae type b).
