# Supplementary material for: Reduction in Severity of All-Cause Gastroenteritis Requiring Hospitalisation in Children Vaccinated against Rotavirus in Malawi
Source: Viruses. 2021 Dec 13;13(12):2491. doi: 10.3390/v13122491 (PMC8707889; doi:10.3390/v13122491)
Supplement: Supplementary file 1 [file viruses-13-02491-s001.zip › Supplementary_Data S7_Figure_S2_Viruses_Submission_Proofed.pdf]

A B

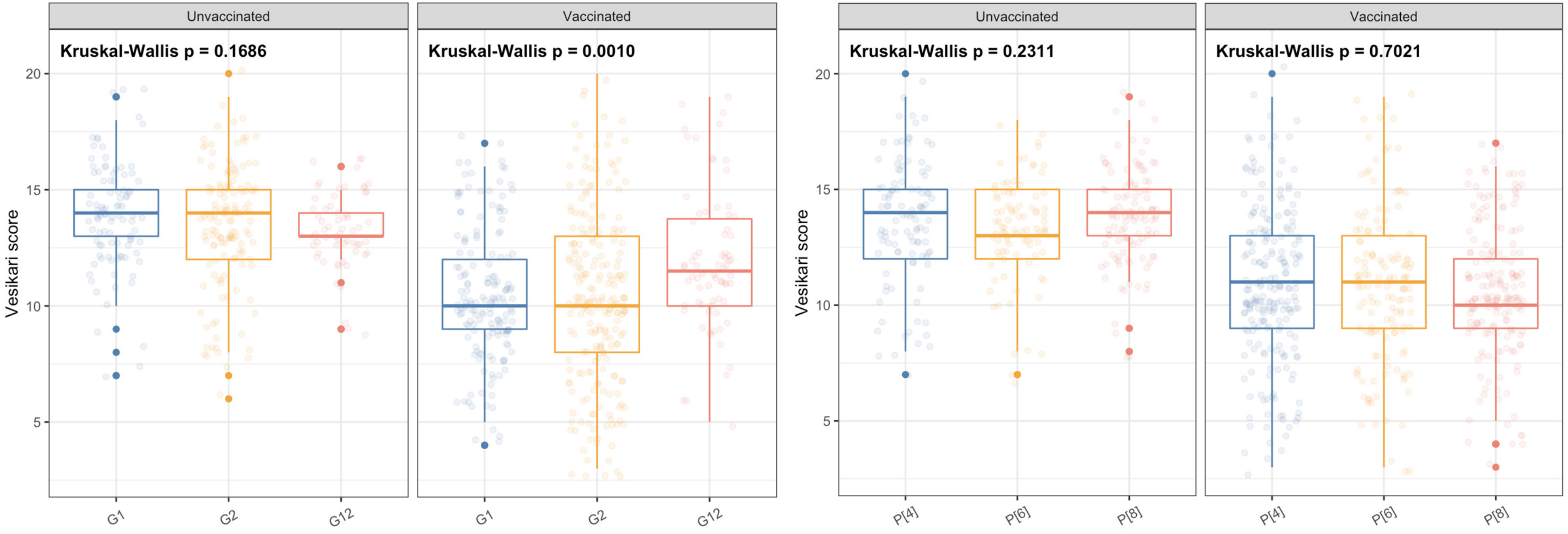

Supplementary Data S7, Figure S2. Severity scores in hospitalised children with rotavirus confirmed gastroenteritis at Queen Elizabeth Central Hospital, Blantyre, Malawi. (A) Vesikari scores in Rotarix®-vaccinated and Rotarix®-unvaccinated children when only rotavirus G genotypes were considered. (B) Vesikari scores in Rotarix®-vaccinated and Rotarix®-unvaccinated children when only rotavirus P genotypes were considered. Vesikari scores = Ruuska and Vesikari scores.
